# Exploring the Application of Bifunctional Metal Chelators in Treating Triple-Negative Breast Cancer

**DOI:** 10.3389/fbioe.2021.697862

**Published:** 2021-08-03

**Authors:** Kuo Li, Youjiu Zhang, Xiaomei Wang, Ran Zhu, Changsheng Ma, Rui Hu

**Affiliations:** ^1^Department of Radiotherapy, Shandong Cancer Hospital and Institute, Shandong First Medical University and Shandong Academy of Medical Sciences, Jinan, China; ^2^School of Radiation Medicine and Protection, Soochow University, Suzhou, China; ^3^Department of Radiation Oncology, Suzhou Municipal Hospital, Suzhou, China

**Keywords:** triple negative breast cancer, bifunctional chelator, cetuximab, targeted internal irradiation therapy, HOPO

## Abstract

**Purpose:** In this study, we independently synthesised and labelled a novel bidentate bifunctional chelating agent, ^177^Lu-3,4-HOPO-Cetuximab, that achieved tight binding between targeting and radioactivity, and evaluated its targeted killing ability of cells *in vitro* and *in vivo*.

**Method:** 3,4-HOPO was successfully synthesised through a series of chemical steps using malt phenol as the raw material, which was then coupled with Cetuximab labelled with ^177^Lu. ^177^Lu-3,4-HOPO-Cetuximab and ^177^Lu-DOTA-Cetuximab was tested for its cell viability and cell-binding rate after different times and at different doses by CCK-8 and cell-binding experiments. ^177^Lu-3,4-HOPO-Cetuximab (~500 μCi) and ^177^Lu-DOTA-Cetuximab (~500 μCi) were injected into the tail vein of a subcutaneous metastasis mouse model of triple-negative breast cancer with a single injection, and tumour volume growth and body weight changes were regularly monitored for 20 days. The radioactivity distribution in nude mice was analysed after sacrifice, and the treated and untreated tumour tissues were analysed by HE staining.

**Result:** The cell viability of ^177^Lu-3,4-HOPO-Cetuximab declined exponentially after treatment for 48 h at 50 Bq/mL to 500 kBq/mL, respectively; the cell activity was slowed down from 8 to 96 h at a dose of 500 kBq; while the binding rate of 4T1 cells in ^177^Lu-3,4-HOPO-Cetuximab from 1 to 24 h, respectively, increased logarithmically, which was similar with ^177^Lu-DOTA-Cetuximab. After 20 days of treatment, the body weight of nude mice with ^177^Lu-3,4-HOPO-Cetuximab and ^177^Lu-DOTA-Cetuximab were hardly changed, while the body weight with physiological saline decreased significantly. The tumour inhibition rate of the ^177^Lu-3,4-HOPO-Cetuximab and ^177^Lu-DOTA-Cetuximab were (37.03 ± 11.16)% and (38.7 ± 5.1)%; HE staining showed that tumour cells were affected by the action of ^177^Lu causing necrosis.

**Conclusion:** The experiments showed that ^177^Lu-3,4-HOPO-Cetuximab has a certain targeted therapeutic ability for triple-negative breast cancer, and it is expected to become a potential targeted nuclear medicine treatment for triple-negative breast cancer.

## Introduction

The Transitional Breast Cancer Research Consortium (TBCRC) conducted a clinical trial (Cleere, 2010; Lisa et al., 2012; Fatima et al., 2015; Chen et al., 2017; Zimei and Zan, 2017; Pindiprolu et al., 2018) and found that although TNBC patients tolerate Cetuximab well with small adverse reactions, its efficacy is limited. This suggests that the combination of Cetuximab and EGFR inhibitor therapy is expected to be a development point for TNBC. Studies have found that EGFR ligands can drive tumour proliferation in an autocrine/paracrine manner, and Cetuximab may block ligand-receptor binding (Wang et al., 2019; Fasano et al., 2021). The studies also confirmed the ability of ^131^I-labelled targeting molecules to target the nuclides of TNBC *via* EGFR.

Over the past few years, new ^177^Lu-labelled radiopharmaceuticals have been researched and developed internationally. Several peptides and monoclonal antibodies (mAbs) have been conjugated to bifunctional chelators and the labelled radionuclide ^177^Lu. Ramli et al. (2011) successfully prepared radioimmunoassays based on the anti-HER-2 monoclonal antibody compound ^177^Lu-DOTA-trastuzumab, which showed potent anticancer effects.

With the development of nuclear medicine, the new bifunctional chelator-HOPO has been discovered by scientists and has begun to enter the field of scientific research, and some HOPO compounds have reached the experimental stage before application. Although DTPA (Stanisz and Henkelman, 2015), DOTA (Eigner et al., 2013), and NOTA (Adam et al., 2007), etc., have shown strong capabilities, research on HOPO-based chelators that link targeting molecules to lanthanide and lanthanide metal ions in nuclear medicine is gaining increasing attention (Cilibrizzi et al., 2018). Deferiprone (1,2-dimethyl-3-hydroxy-4-pyridone, DFP) has been approved in Europe as an effective iron removal agent as a bidentate 3,4-HOPO.

Previous studies in our group have proved that ^131^I-labelled cetuximab is easy to fall off after injection in mice for a period of time. Based on the above research background, this experiment independently prepared a novel bifunctional chelating agent, 3,4-HOPO, using 3,4-HOPO as a bifunctional chelating agent for ^177^Lu-labelled Cetuximab to investigate the effects of ^177^Lu-3,4-HOPO-Cetuximab in the treatment of TNBC.

## Experimental Materials

### Experiment Equipment

Wantong titrator (Swiss Wantong China Ltd., Switzerland); FJ-391A4 radioactivity metre (Beijing Nuclear Instrument Factory); LB 2111 Multi Crystal Gamma Counter (Berthold Technologies, Germany); KDC-20 low-speed centrifuge (Hefei Keda Innovation Co., Ltd. Zhongjia Branch).

### Cell Lines and Experimental Animals

The nude mouse triple-negative breast cancer cell line 4T1 cells were provided by Soochow university laboratory. Female BALB/c nude mice were purchased from Nanjing Skrui Biotechnology Co., Ltd., aged 4–6 weeks, and were raised at the Experimental Animal Centre of Suzhou University according to SPF requirements and approved by the animal ethics committee of Soochow University.

### Laboratory Supplies

3-hydroxy-2-methyl-4-pyrone (maltophenol, 99%, Jingda, Anhui Jinhe Industrial Co., Ltd.); DOTA-NHS purchased from MACROCYCLICS, Europe; Cetuximab Purchased from Shanghai Saima Biotech Co., Ltd.; ^177^LuCl_3_ was purchased from ITG Germany.

## Methods

### Synthesis of Bidentate 3,4-HOPO

**Figure 1:**
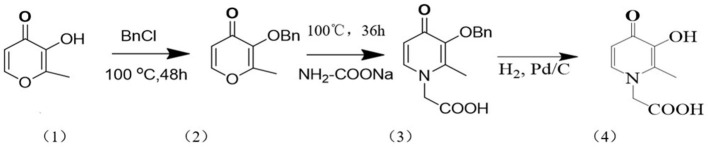
Schematic diagram of the synthesis process of 3, 4-HOPO.

The synthesis process of bidentate 3,4-HOPO is shown in Figure 1.

Accurately weigh 43 g of maltol (1) dissolved in 285 mL of methanol and 35 mL of 10.25 mol/L NaOH solution, add 45 mL of benzyl chloride, and reflux at 100°C for 48 h to obtain orange oily liquid benzyl maltol (2).

Take benzyl maltol (2) 73.23 g dissolved in 210 mL mixed solution of methanol and water (volume ratio 1:1), add sodium glycinate powder, reflux reaction at 100°C for 36 h, then extract with 40–100 mL dichloromethane, water phase The concentrated HCl (12 mol/L) was adjusted to a pH 2, and the precipitated yellow crystals were filtered and dried in a vacuum oven for 30 h to obtain the product Bn-3,4-HOPO (3).

After the Bn-3,4-HOPO was dissolved in methanol, a certain amount of palladium carbon (about 5% by mass of the reactant) was weighed and added slowly to the reaction solution, and hydrogen was introduced and stirred for 4 h. After completion of the reaction, the mixture was filtered to obtain palladium carbon and a product mixture; after adding DMF (N,N-dimethylformamide), the mixture was heated and stirred at 80°C until the solution became a black suspension, and the filtered filtrate was steamed to obtain rice. The white solid was dried in vacuo to give the product as 3,4-HOPO (4). The results of the nuclear magnetic resonance test were as follows: 1H NMR (400 MHz, DMSO): δ 7.53 (d, J = 7.2 Hz, 1H), 6.11 (d, J-7.2 Hz, 1H), 4.82 (s, 2H), 2.15 (s, 3H); LC-MS [M + H+] m/z: 183.87.

### Preparation of 3,4-HOPO-Cetuximab and DOTA-Cetuximab (Hereinafter Referred to as 3,4-HOPO-Ab and DOTA-Ab) Solution

Take 200 μL of Ab solution from the −80°C refrigerator and thaw it in a water bath. The ratio of 3,4-HOPO:NHS = 1:1.2 in 0.25 mol/L CH_3_COONH_4_ solution for 5 min, add 3,4- HOPO or DOTA ratio 1.2 equivalent EDC continued to activate for 30 min; molar ratio 3,4-HOPO or DOTA: Ab = 50:1 coupling; addition of 0.25 mol/L CH_3_COONH_4_ solution to 0.5 mL; constant temperature mixing at room temperature for 24 h to complete coupling Union. The coupled 3,4-HOPO-Ab solution or DOTA solution was centrifuged at 4,500 rpm for 45 min at room temperature, followed by ultrafiltration at 6,000 rpm for 15 min; the conjugate was collected, 0.5 mL of 0.25 mol/L CH_3_COONH_4_ solution was added, and placed. Store this at 4°C.

### Preparation of ^177^Lu-3,4-HOPO-Ab and ^177^Lu-DOTA-Ab Solution

Remove 350 μL of purified 3,4-HOPO-Ab solution or ^177^Lu-DOTA-Ab solution from a 4°C refrigerator; add 20 μL of ^177^LuCl_3_ dilution solution to the 3,4-HOPO-Ab solution or ^177^Lu-DOTA-Ab solution (activity is about 1 mCi); add CH_3_COONH_4_ buffer with pH 5. The solution was 500 μL; the mixture was shaken for 30 min at 37°C under a vortex constant temperature mixer to complete the labelling. The reaction solution after the completion of the labelling was centrifuged at 4,000 rpm for 15 min at room temperature; the buffer was discarded, the label was rinsed from the inner liner with a 0.25 mol/L CH_3_COONH_4_ solution, and the collected liquid was stored in an EP tube. The radiochemical purity was measured by a TLC method in a test tube in which the developing solvent was acetic acid: ammonium acetate (pH 3). The labelling rates of the ^177^Lu-3,4-HOPO-Ab and ^177^Lu-DOTA-Ab solutions were (89.7 ± 0.9)% and (92.6 ± 1.0)%. After purification, the radiochemical purity were both higher than 95%.

### Cell-Binding Rate of ^177^Lu-Ab and ^177^Lu-3,4-HOPO

When the cells grow to more than 90%, the cell concentration is adjusted to about 1.0 × 10^5^/mL, and 1 mL is added to each well in the 12-well plate and cultured overnight in the cell incubator at 37°C. When the cells adhered to the logarithmic growth phase on the second day after administration, 0.37 kBq markers were added to each hole. After 1, 2, 4, 8, and 24 h, the supernatant was added to the supernatant tube and washed twice by PBS, the cleaning fluid was also added to the supernatant tube; the cells were digested with trypsin, and the cell suspension was inhaled into the cell tube, washed with PBS for 2 times, and the radioactivity count (B) of the cell tube and the radioactivity count (F) of the supernatant tube were measured by γ counter. The cell-binding rate of the marker is B/(B+F) × 100%.

### Effect of CCK-8 on the Proliferation of 4T1 Cells by ^177^Lu

4T1 cells were uniformly inoculated into 96-well plates and the number of cells was 8,000/well. The cells were placed in a 37°C, 5% CO_2_ saturated humidity incubator; the cells were fully attached for about 24 h. Six sets of parallel wells were randomly selected from each group, the medium was discarded; the medium containing ^177^LuCl_3_, ^177^Lu-3,4-HOPO-Ab, and ^177^Lu-DOTA-Ab was, respectively, added to a 96-well plate by 50 Bq/mL~ 500 kBq/mL, 100 μL per well, the medium containing 2 μg/mL of Ab was a positive control group and the blank control group was drug-free. After 4 h, replace the drug with a drug-free solution, and re-dispose it in a 37°C, 5% CO_2_ saturated humidity incubator. After 48 h, discard the medium, and add 100 μL CCK-8 per well. About 0.5 h until the culture medium showed an orange colour, and the absorbance was measured by a microplate reader.

Afterwards, 100 μL of ^177^LuCl_3_, ^177^Lu-3,4-HOPO-Ab, and ^177^Lu-DOTA-Ab with a radioactive dose of 500 kBq/mL were added to a 96-well plate and then placed at 37°C, 5% CO_2_ saturated humidity. After the box was cultured for 4 h, the culture solution was discarded, the drug-free culture solution was replaced, and it was then discarded in the incubator for 8, 24, 48, 72, and 96 h, respectively. In total, 100 μL of medium containing 10% CCK-8 was added to each well. About 0.5 h until the culture medium showed an orange colour and was placed on a microplate reader. Cell viability (%) = [A (dosing) − A (blank)]/[A (control) − A (blank)]; A (dosing) is the absorbance of wells with cells, CCK-8 solution and drug solution; A (blank) is the absorbance of wells with medium and CCK-8 solution without cells; A (control) is the absorbance of wells with cells and CCK-8 solution without drug.

### Changes in the Binding Rate of ^177^Lu-3,4-HOPO-Ab to 4T1 Cells Over Time

After the cells were grown to more than 90% of the digested cells, the adjusted cell concentration was about 1.0 × 10^5^ cells/mL, and 1 mL was added to each well of a 12-well plate, and cultured in a 37°C cell culture incubator overnight. When the cells were attached to the next day and were in the logarithmic growth phase, 1 mL of the culture medium containing the radioactive label was replaced (about 0.37 kBq per well), and three replicate wells were set in each group. After the markers were treated with 4T1 cells for 1, 2, 4, 8, and 24 h, the supernatant was aspirated and transferred to the supernatant EP tube. After washing twice with PBS, the washing solution was also incorporated into the supernatant EP tube. The cells were digested with a trypsin digestion solution. After the cells were completely digested, the cell suspension was inhaled into the EP tube of the cells, washed twice with PBS, and the washing solution was also added to the EP tube, and the cells were separately measured in a γ counter. The radioactivity count of the tube (B) and the radioactivity count of the supernatant tube (F). The cell-binding rate of the marker was B/(B+F) × 100%.

### Tumour Growth Inhibition Experiment

In total, 30 nude mice bearing tumours with a diameter of ~0.4–0.6 cm were randomly divided into two groups (treatment group and control group). There was no significant difference in body weight between the two groups.

In the treatment group, ^177^Lu-3,4-HOPO-Ab was injected into the tail vein at 100 μL/mouse (~500 μCi), and the control group was injected with an equal volume of normal saline *via* the tail vein. Treatment was administered once. After the completion of the tail vein injection, the state and activity of the nude mice were observed every day. The nude mice were weighed on day 0, day 3, day 5, day 7, day 10, and day 20 after dosing. Additionally, the long diameter and short diameter of each group of tumours were measured with a Vernier calliper, and the volume of the tumour was calculated for a total of 20 days.

The calculation method of the tumour volume was as follows:

$$\text{V=}\frac{\text{X}\times {\text{Y}}^{\text{2}}\;}{\text{2}},$$

where X is the long diameter and Y is the short diameter.

The tumour growth inhibition rate (i.e., tumour inhibition rate) is the tumour inhibition rate = [(A − B)/A] × 100%, where A is the control group and B is the treatment group.

On the 20th day after administration, blood was collected from the retro-orbital sinus, and the nude mice were sacrificed. The nude mice were dissected, and organs such as the heart, liver, spleen, lung, kidneys, stomach, intestine, tumour, bone, muscle, and skin were taken, weighed, and detected by radioactive gamma count. The result was converted to %ID/g [%ID/g = radioactive count of the organ/(radioactive count rate of the injected drug × organ mass)], and the gamma counter was calibrated to be 1.2 × 10^6^ cpm = 1 μCi.

### Immunohistochemical Detection of Tumour Tissue Before and After Treatment

4T1 tumour tissues were extracted without treatment or with treatment, fixed by 4% paraformaldehyde for 24 h. HE staining and immunohistochemical analysis were entrusted to Wuhan Sevier Biotechnology Co., Ltd. The rate of EGFR positive cells is the average number of positive cells in 3 visual fields (200×) of a certain type of cells: 0–5% is grade 0, 6–25% is grade 1, 26–50% is grade 2, 51–75% is grade 3, >75% is grade 4.

### Statistical Analysis

The experimental results were expressed as mean ± standard deviation (x̄ ± s). The data were analysed by *SPSS 19.0* software. One-way analysis of variance (ANOVA) was performed in a completely randomised design. *P* < 0.05 was considered statistically significant.

## Results

### Analysis of 3,4-HOPO

**Figure 2:**
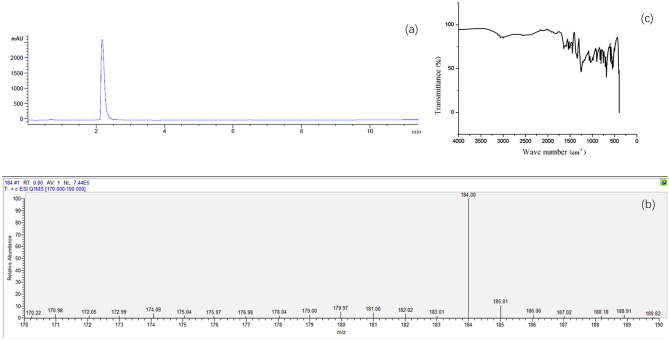
HPLC **(a)**, mass spectrometric chromatogram **(b)** and infrared spectrum **(c)** of 3,4-HOPO.

The results of UV absorption at 254 nm showed that the HPLC chromatogram of 3,4-HOPO occurred an absorption peak only at 2.2 min with no obvious impurity peaks appeared elsewhere. The purity of 3,4-HOPO is 98.6% (Figure 2a). Mass spectrometry (Figure 2b) shows the LC-MS [M+H^+^] m/z: 184.00. Since it is the test result in H^+^ mode, it is inferred that the actual molecular weight is 183.00 Infrared spectrum (Figure 2c) ensured that the structure of 3,4-HOPO is correct.

**Figure 3:**
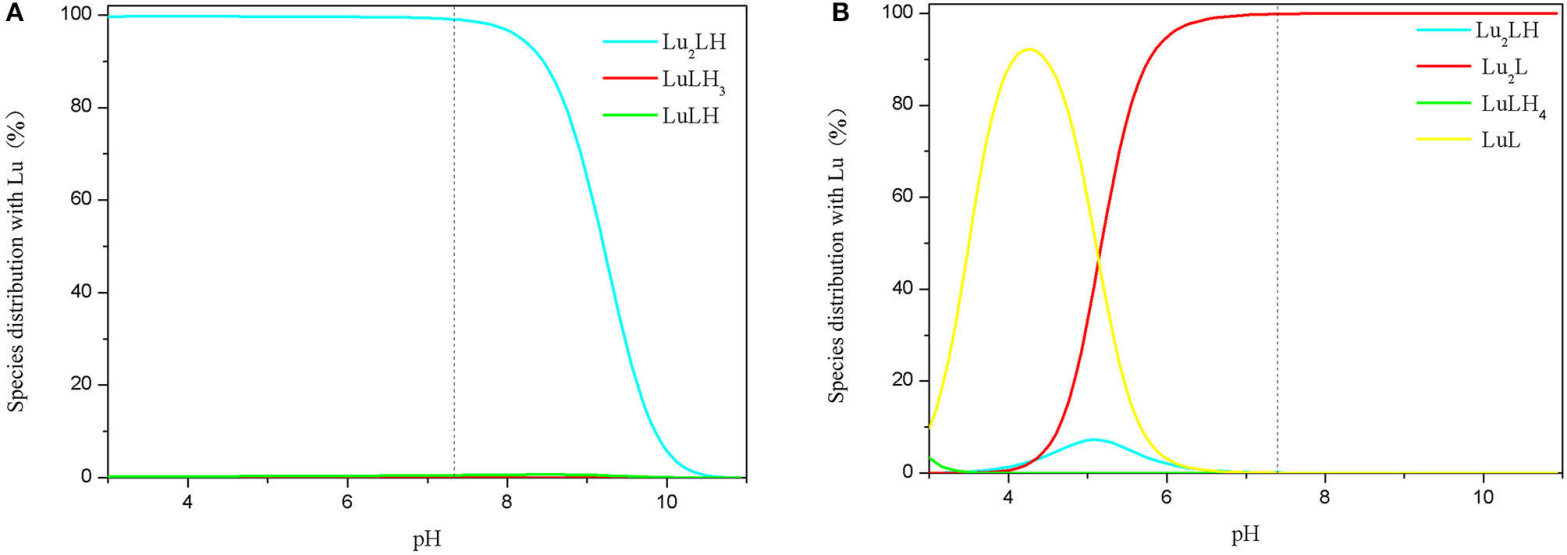
Species distribution of 3,4-HOPO **(A)** and DOTA **(B)** with Lu^3+^ complexes (initial concentrations of Lu^3+^ ions and 3,4-HOPO were 1 × 10^−4^ M and 1 × 10^−3^ M, respectively).

As shown in Figure 3. The dissociation constants pK_a1_ and pK_a2_ of 3,4-HOPO are 10.1 ± 0.1 and 3.7 ± 0.1, respectively. The results show that 3,4-HOPO reaches the dissociation equilibrium at pH = 10.1; the second-order dissociation constant pK_a2_ (3.7) indicates that 3,4-HOPO will be protonated at low pH and reach a deprotonation equilibrium at pH = 3.7.While the dissociation constants pKa1, pKa2, pKa3, pKa4, and pKa5 of DOTA are 2.5 ± 0.2, 2.5 ± 0.2, 4.6 ± 0.2, 8.7 ± 0.2, and 10.1 ± 0.2, respectively. The results showed that DOTA reached the first-order and second-order dissociation equilibrium at pH = 2.5, the third-order dissociation equilibrium at pH = 4.6, the fourth-order dissociation equilibrium at pH = 8.7, and the fifth-order dissociation equilibrium at pH = 10.1.

The complexation behaviours of 3,4-HOPO and Lu^3+^ ions were studied by potentiometric titration. The cumulative formation constants of logβ_0lh_ and pM were obtained by *Hyperquad 2008* as shown in Table 1. The distribution of species in the range of pH 3–11 was obtained with *Hyss 2009*. The maximum enrichment zone of chelators with Lu^3+^ (enriched density above 95%) was pH < 8.2 in the form of Lu_2_LH for 3,4-HOPO and pH < 6.0 in the form of Lu_2_L for DOTA, respectively.

**Table 1:** Complexation constants of 3,4-HOPO and DOTA with Lu^3+^.

**Metal ion**	**Coordination material**					
	**3,4-HOPO**			**DOTA**		
	**m l h**	**Log*β*_*mlh*_**	**pM**	**m l h**	**Log*β*_*mlh*_**	**pM**
Lu^3+^			15.3			13.6
	111	16.9 ± 0.1		114	23.6 ± 0.5	
	211	26.6 ± 0.1		110	15.3 ± 0.3	
	210	11.4 ± 0.1		211	30.1 ± 1.2	
				210	21.0 ± 0.2	

*m represents coordination metal ion, l represents chelate, h represents hydrogen ion; pM = –log[m_free_]*.

### Effect of ^177^Lu Labelling Material on Proliferation of 4T1 Cells

**Figure 4:**
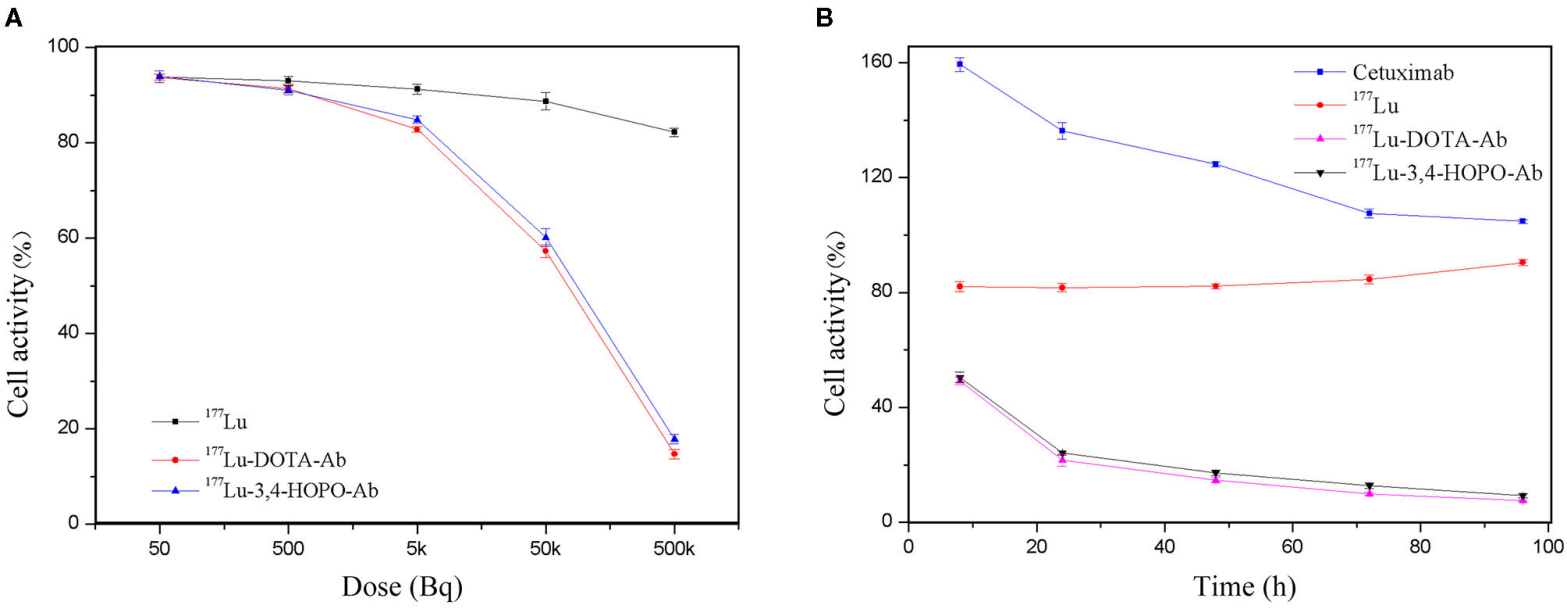
Effect of ^177^Lu-DOTA-Ab and ^177^Lu-3,4-HOPO-Ab on the activity of 4T1 cells **(A)** is the effect with dose and **(B)** is the effect with time.

The effect of ^177^Lu-DOTA-Ab and ^177^Lu-3,4-HOPO-Ab on the proliferation of 4T1 cells is shown in Figure 4A and Table 2. ^177^Lu has a certain inhibitory effect on cell proliferation. With the increase of dose (Figure 4A and Table 2), the cell activity changes from (93.85 ± 1.2)% of 50 Bq/mL to (82.2 ± 0.87)% of 500 kBq/mL for ^177^Lu. However, the ^177^Lu-3,4-HOPO-Ab and ^177^Lu-DOTA-Ab have an absolute inhibitory effect on cell proliferation, the cell activity changes from (93.91 ± 1.24)% and (93.75 ± 0.64)% of 50 Bq/mL to (17.82 ± 1.03)% and (14.70 ± 0.99)% of 500 kBq/mL. Compared with ^177^Lu, the ^177^Lu-3,4-HOPO-Ab had a statistically significant difference in cell viability at a dose of 500 Bq/mL (*P* < 0.05), while the dose was increased to 5 kBq/mL, the difference of cell activity was statistically significant (*P* < 0.05) between ^177^Lu and ^177^Lu-3,4-HOPO-Ab or ^177^Lu-DOTA-Ab.

**Table 2:** The effect of ^177^Lu-DOTA-Ab and ^177^Lu-3,4-HOPO-Ab on the activity of 4T1 cells with the dose increase.

**Medicine**	**Dose (Bq/mL)**				
	**50**	**500**	**5k**	**50k**	**500k**
^177^Lu	93.85 ± 1.20	92.95 ± 0.92	91.25 ± 1.06	88.70 ± 1.84	82.20 ± 0.87
^177^Lu-DOTA-Ab	93.75 ± 0.64	91.35 ± 0.92	82.80 ± 0.58^&^	57.25 ± 1.37^&^	14.70 ± 0.99^&^
^177^Lu-3,4-HOPO-Ab	93.91 ± 1.24	91.01 ± 0.92^&^	84.84 ± 0.74^#^^&^	60.15 ± 1.84^&^	17.82 ± 1.03^#^^&^

& *Means the difference was statistically significant (P < 0.05) compared with the ^177^Lu*,

# *Means ^177^Lu-DOTA-Ab has the significant difference (P < 0.05) compared with the ^177^Lu-DOTA-Ab*.

As shown in Figure 4B and Table 3, Cetuximab promoted cell proliferation over a short period of time. With the increase of time, the cell activity decreased from (159.37 ± 2.46)% at 8 h to (104.75 ± 0.70)% at 96 h after replacement of drug-free medium. The ^177^Lu at 500 kBq and cell culture for 8 h had a slight inhibitory effect on cell proliferation. With the increase in time, the cell activity gradually increased after the initial plateau, it was from (82.10 ± 1.75)% after 8 h of drug-free medium replacement to (90.40 ± 1.12)% at 96 h. However, ^177^Lu-DOTA-Ab and ^177^Lu-3,4-HOPO-Ab were cultured for 8 h had a good inhibitory effect on cell proliferation. With the increase of time, the cell activity decreased continuously (49.20 ± 1.40)% for ^177^Lu-DOTA-Ab and (50.47 ± 1.83)% for ^177^Lu-3,4-HOPO-Ab at 8 h after replacing the drug-free medium were changed to (7.50 ± 0.28)% and (9.24 ± 0.68)% at 96 h. And the differences at each time point were statistically significant (*P* < 0.05) compared with the cell activity at 8 h, we also found that ^177^Lu-DOTA-Ab had a better therapeutic effect than ^177^Lu-3,4-HOPO-Ab from 48 to 72 h, but the difference was not statistically significant (*P* > 0.05) at 96 h between ^177^Lu-DOTA-Ab and ^177^Lu-3,4-HOPO-Ab.

**Table 3:** The effect of ^177^Lu-DOTA-Ab and ^177^Lu-3,4-HOPO-Ab on the activity of 4T1 cells with the time increase.

**Medicine**	**Time (h)**				
	**8**	**24**	**48**	**72**	**96**
Cetuximab	159.37 ± 2.46	136.24 ± 2.83	124.63 ± 0.84	107.52 ± 1.63	104.75 ± 0.70
^177^Lu	82.10 ± 1.75	81.70 ± 1.42	82.20 ± 0.87	84.50 ± 1.53	90.40 ± 1.12^&^
^177^Lu-DOTA-Ab	49.20 ± 1.40^%^	21.60 ± 2.19^&^^%^	14.70 ± 0.99^&^^%^	9.90 ± 0.49^&^^%^	7.50 ± 0.28^&^^%^
^177^Lu-3,4-HOPO-Ab	50.47 ± 1.83^%^	24.18 ± 0.74^&^^%^	17.82 ± 1.03^#^^&^^%^	12.75 ± 0.91^#^^&^^%^	9.24 ± 0.68^&^^%^

% *Means ^177^Lu-DOTA-Ab and ^177^Lu-3,4-HOPO-Ab had significant difference (P < 0.05) compared with the ^177^Lu*,

# *Means ^177^Lu-3,4-HOPO-Ab had significant difference (P < 0.05) compared with the ^177^Lu-DOTA-Ab, and*

& *Means the difference was statistically significant (P < 0.05) compared with 8 h in treatment groups*.

### 4T1 Cell-Binding Rate of ^177^Lu-3,4-HOPO-Ab

**Figure 5:**
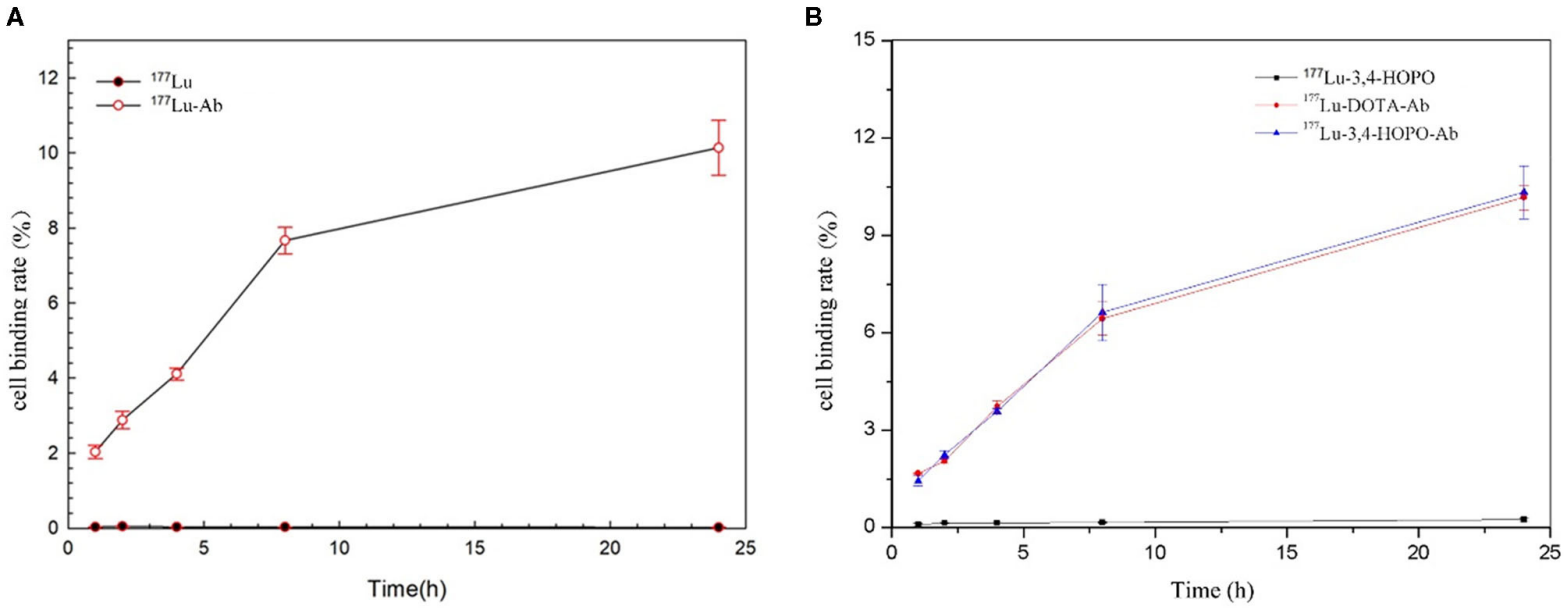
The time-varying curve of the 4T1 cell-binding rate of ^177^Lu and chelates. **(A)** shows the 4T1 cell-binding rate with or without Cetuximab, **(B)** shows the ^177^Lu-DOTA-Ab, and ^177^Lu-3,4-HOPO-Ab could have a better effect for 4T1 cell binding.

The results showed that 4T1 cell was no binding effect with ^177^Lu-3,4-HOPO and ^177^Lu in Figure 5 and Table 4, while the binding rate of Cetuximab to cells increased with time. But after reaching a certain time, the growth rate decreased, and could not achieve the effect of ^177^Lu-3,4-HOPO-Ab and ^177^Lu-DOTA-Ab.

**Table 4:** Time-varying values of 4T1 cell-binding rate of ^177^Lu and chelates.

**Medicine**	**Time (h)**				
	**1**	**2**	**4**	**8**	**24**
^177^Lu-3,4-HOPO	0.087 ± 0.040	0.142 ± 0.025	0.147 ± 0.013	0.159 ± 0.015	0.237 ± 0.042
^177^Lu-DOTA-Ab	1.67 ± 0.02^*^	2.06 ± 0.08^*^	3.72 ± 0.19^*^	6.44 ± 0.51^*^	10.17 ± 0.37^*^
^177^Lu-3,4-HOPO-Ab	1.45 ± 0.17^*^	2.24 ± 0.12^*^	3.58 ± 0.09^*^	6.63 ± 0.87^*^	10.33 ± 0.82^*^

* *Means the difference was statistically significant (P <0.05) compared with the cell-binding rate of ^177^Lu-3,4-HOPO*.

The change of the binding rate of ^177^Lu-3,4-HOPO-Ab and ^177^Lu-DOTA-Ab to 4T1 cells with time is shown in Figure 5B. It can be seen that the cell-binding rate is positively correlated with the time change, showing a certain targeting ability of the antibody. The binding rate of 4T1 cell with ^177^Lu-3, 4-HOPO-Ab, and ^177^Lu-DOTA-Ab were (1.45 ± 0.17)% and (1.67 ± 0.08)% at 1 h, increased to (10.33 ± 0.82)% and (10.17 ± 0.46)% at 24 h. There were statistically significant differences (*P* < 0.05) in the cell-binding rate between ^177^Lu-3,4-HOPO and ^177^Lu-3,4-HOPO-Ab as well as ^177^Lu-DOTA-Ab all the time.

### *In vivo* Experiment of ^177^Lu Labelled Material on 4T1 Nude Mice

**Figure 6:**
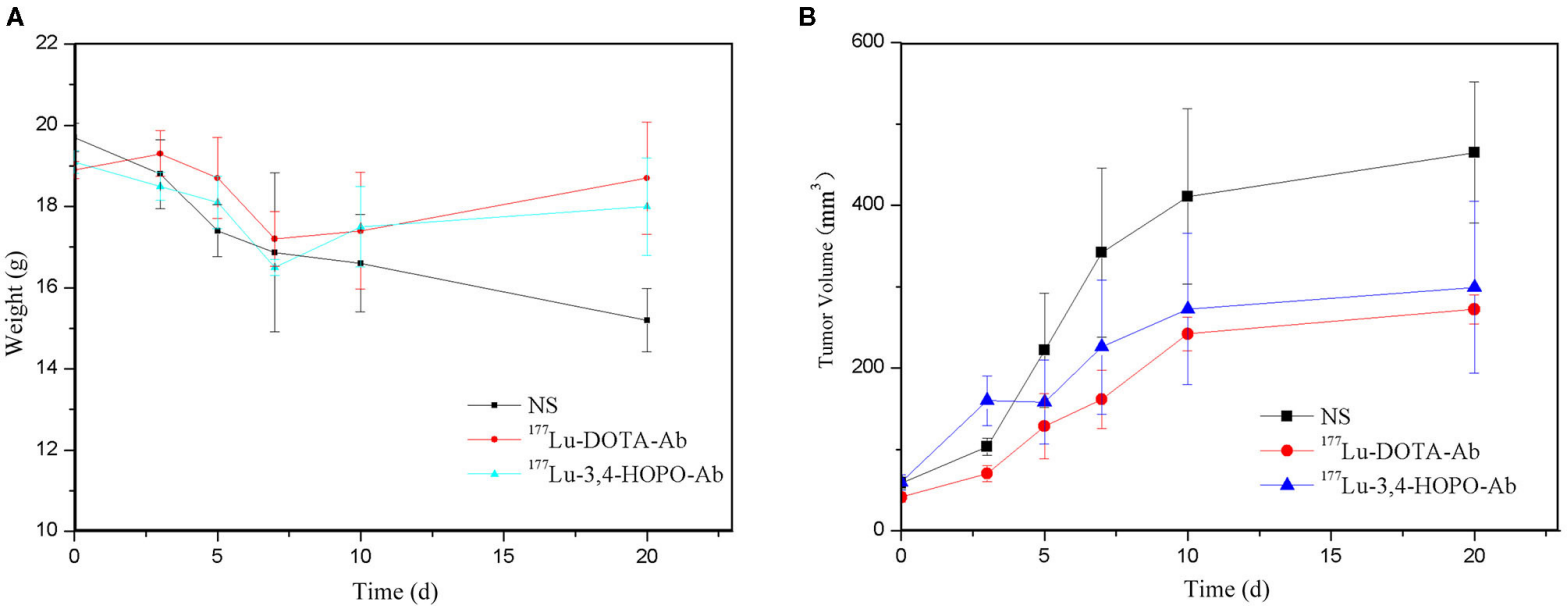
Body weight and tumour volume changes in nude mice treated over time (NS is physiological saline). **(A)** shows the changes of body weight with time and **(B)** shows the change of tumor volume with time.

The body weight changes of the 4T1 tumour-bearing nude mice in physiological saline, ^177^Lu-DOTA-Ab, and ^177^Lu-3,4-HOPO-Ab over time after administration are shown in Figure 6A and Table 5. Before administration, the weight of the nude mice in the saline, ^177^Lu-DOTA-Ab, and ^177^Lu-3,4-HOPO-Ab were (19.74 ± 0.35) g, (18.9 ± 0.21) g, and (19.11 ± 0.28) g, respectively, with the difference between each group were not statistically significant (*P* > 0.05). After a period of feeding, the weight change trend of the ^177^Lu-labelled monoclonal antibody treatment groups was increased, while it was decreased in the saline group. After 20 days, the weights of nude mice with ^177^Lu-3,4-HOPO-Ab and ^177^Lu-DOTA-Ab were (18.02 ± 1.20) g and (18.7 ± 1.38) g, (15.21 ± 0.78) g in saline, and there were statistically significant differences (*P* < 0.05) between the treatment groups and the saline group from the beginning at 7th day.

**Table 5:** Weight in nude mice treated over time.

**Group**	**Time (△)**					
	**0**	**3**	**5**	**7**	**10**	**20**
NS	19.7 ± 0.4	18.8 ± 0.8	17.4 ± 0.6	16.9 ± 1.9	16.6 ± 1.2	15.2 ± 0.8
^177^Lu-DOTA-Ab	18.9 ± 0.2	19.3 ± 0.6	18.7 ± 1.0	17.2 ± 0.7	17.4 ± 1.4^*^	18.7 ± 1.4^*^
^177^Lu-3,4-HOPO-Ab	19.1 ± 0.3	18.5 ± 0.4	18.1 ± 0.6	16.5 ± 0.2	17.5 ± 1.0^*^	18.0 ± 1.2^*^

* *Means the difference was statistically significant (P <0.05) between treatment groups and NS (physiological saline) group*.

As shown in Figure 6B and Table 6. Before the start of treatment, the volume of 4T1 xenografts in saline, ^177^Lu-DOTA-Ab, and ^177^Lu-3,4-HOPO-Ab were (58.60 ± 7.53) mm^3^, (51.2 ± 6.3) mm^3^, and (59.64 ± 9.60) mm^3^, respectively, and there were no differences (*P* > 0.05) between each of two groups. After 20 days of treatment, the volume of tumours in ^177^Lu-3,4-HOPO-Ab and ^177^Lu-DOTA-Ab were (299.20 ± 105.70) mm^3^ and (272.2 ± 17.8) mm^3^, which in saline was (465.04 ± 87.02) mm^3^. There were statistically significant differences (*P* < 0.01) in the volume of transplanted tumours within the treatment groups and the saline group from the beginning of the 7th day. The tumour inhibition rates of ^177^Lu-3,4-HOPO-Ab and ^177^Lu-DOTA-Ab were (37.03 ± 11.16)% and (38.7 ± 5.1)%, respectively. Tumor treatment changes in nude mice is shown in Figure 7.

**Table 6:** Tumour volume values in nude mice treated over time.

**Group**	**Time (d)**					
	**0**	**3**	**5**	**7**	**10**	**20**
NS	58.6 ± 7.5	103.1 ± 10.7	221.9 ± 70.2	341.8 ± 104.1	410.9 ± 107.8	465.0 ± 87.0
^177^Lu-DOTA-Ab	51.2 ± 6.3	70.4 ± 9.8^*^	128.4 ± 39.9	161.3 ± 35.6^*^	241.9 ± 20.5^*^	272.2 ± 47.8^*^
^177^Lu-3,4-HOPO-Ab	59.6 ± 9.6	159.7 ± 30.6^#^	183.6 ± 84.9^#^	225.8 ± 82.5^*^^#^	272.6 ± 93.2^*^	299.2 ± 105.7^*^

* *Means the difference was statistically significant (P < 0.05) between treatment groups and NS (physiological saline) group*.

# *Means the difference was statistically significant (P < 0.05) between the ^177^Lu-3,4-HOPO-Ab group and ^177^Lu-DOTA-Ab group*.

**Figure 7:**
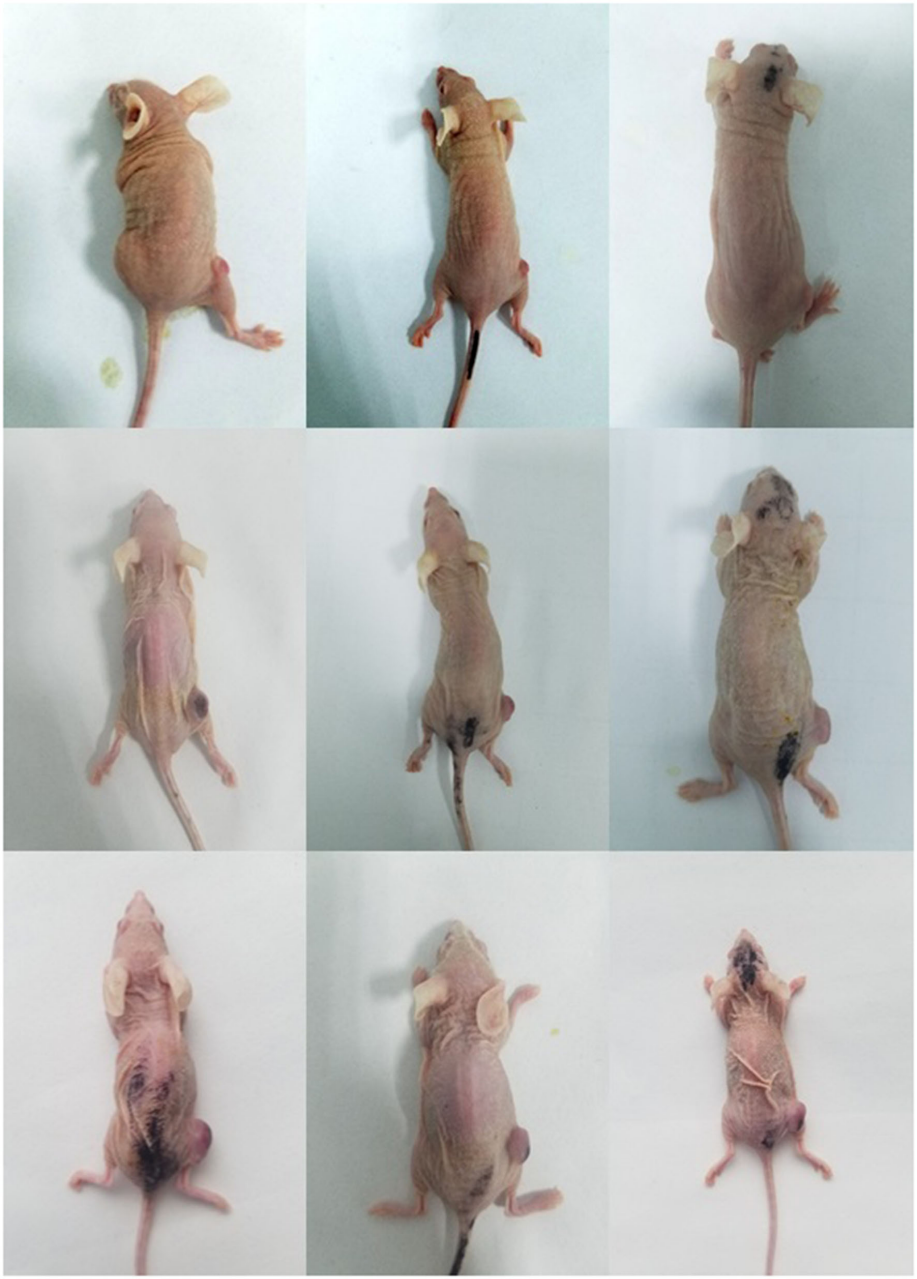
Tumour treatment changes in nude mice between treatment group and control group (lines 1–3 refer to the status of mice on the 0th, 7th, and 20th day after treatment, respectively. Columns 1–3 refer to the status of mice with NS, ^177^Lu-3,4-HOPO-Ab, and ^177^Lu-DOTA-Ab, respectively.

### *In vivo* Radioactivity Distribution in Nude Mice After 20 Days of Administration

**Figure 8:**
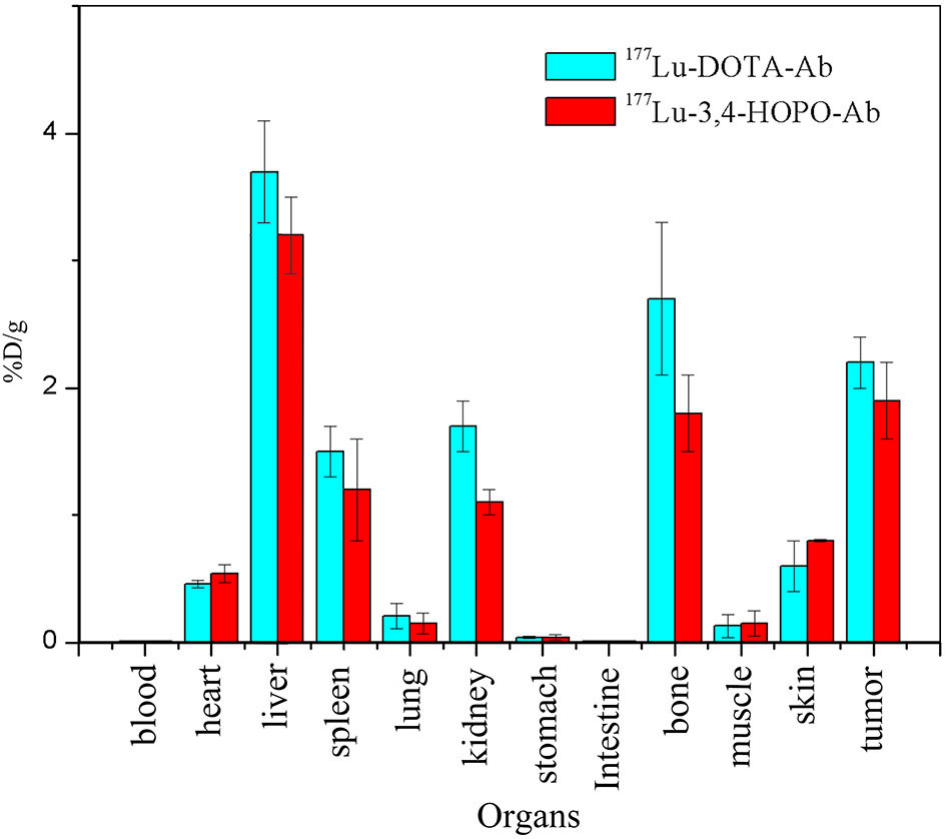
*In vivo* distribution of nude mice after injection for 20 days.

After 20 days of injection in the tail vein, the radioactivity profile of the 4T1 tumour-bearing nude mice (Figure 8) showed that the radioactivity was mainly concentrated in the liver and bone, the ^177^Lu-3,4-HOPO-Ab and ^177^Lu-DOTA-Ab were (3.20 ± 0.30)%ID/g and (3.69 ± 0.42)%ID/g in liver and (1.83 ± 0.30)%ID/g and (2.7 ± 0.6)%ID/g in bone. However, the ^177^Lu-3,4-HOPO-Ab group and ^177^Lu-DOTA-Ab group had radioactivity were, respectively (1.90 ± 0.32)%ID/g and (2.2 ± 0.2)%ID/g in tumours.

### HE Staining and Immunohistochemistry

**Figure 9:**
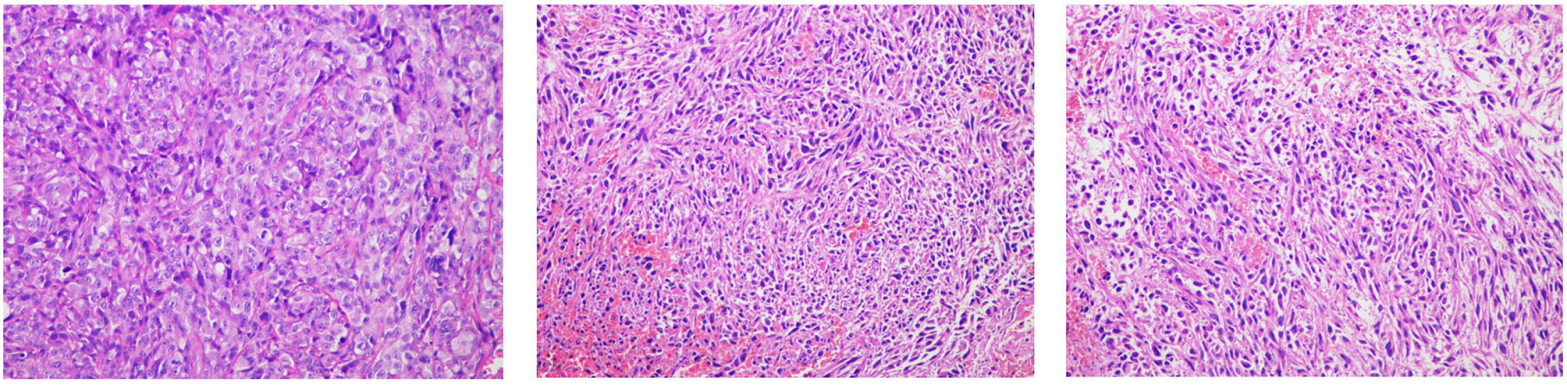
Tumour tissue sections and EGFR immunohistochemistry (10 × 20; the left is the saline control group, middle is the ^177^Lu-DOTA-Ab group, and right is the ^177^Lu-3,4-HOPO-Ab group).

HE staining of ^177^Lu with and without treatment of 4T1 tumour, as shown in Figure 9. HE staining sections of the saline control group (left) showed typical mega-massive tumour cells, see multiple divisions, cell growth was strong, cells were closely arranged, and the ratio of nuclear to cytoplasm was high. Treatment group (mid and right) tumour cells showed partial necrosis and inflammation, and the cells were loosely arranged with a low nuclear-to-plasma ratio.

EGFR is mainly expressed on the cell membrane, and particles ranging from pale yellow to tan are representative of EGFR expression. For the untreated group (left), there was more EGFR expression in the tumour tissues; after treatment (mid and right), the expression of EGFR in the tumour decreased, and the proportion of positive cells was less than that in the control group.

According to the semi-quantitative scoring method, the EGFR positive rate of the untreated group was grade 10. The EGFR positive rate of the ^177^Lu-3,4-HOPO-Ab and ^177^Lu-3,4-HOPO-Ab groups were both grade 2.

## Discussion

Hydroxypyridone (HOPO) is an outstanding building block for the development of various reagents in the field of metal chelation. At present, in-depth analysis of the synthesis methods of HOPO-based ligands and many methods for achieving optimal biological activity have been reported (Ana et al., 2012).

The pH of human body fluid is close to 7.4, so we analysed the species distribution relationship and complexation constant of 3,4-HOPO interacting with different metal ions at pH 7.4. 3,4-HOPO and DOTA in Lu^3+^ system existed in the form of Lu_2_LH (99.0%) and Lu_2_L (99.8). In this state, the complexation constant of 3,4-HOPO is 5 orders of magnitude higher than that of DOTA.

In 1907, the French chemist G. Urbain recrystallised several times with a solution of niobium nitrate, from which a new element “Lutecium” was isolated and named “Paris” in ancient Latin-Lutetia named it Lutetium (Lu) (Yokel et al., 2000). Cetuximab is an FDA-approved monoclonal antibody that targets EGFR cancer cells and inhibits the growth of these cells (Listed, 2006). Cetuximab is radiolabelled with various diagnostic and therapeutic radionuclides by various bifunctional chelators and targeted in preclinical models and clinical settings (Sihver et al., 2014). Cetuximab, among the many therapeutic radionuclides, due to its excellent physical properties and its advantages in the field of tumour therapy, has been proven to be suitable for radioimmunotherapy of tumours, and its research in the field of nuclear medicine internal radiation therapy requires more attention (Kwekkeboom et al., 2003; Feng et al., 2018).

CCK-8 experiments showed that free ^177^Lu had no killing effect for 4T1 cells, while ^177^Lu-3,4-HOPO or ^177^Lu-DOTA conjugated with antibody displayed little damage to cells at a low dose and cell lethality with increasing dose. It also increased exponentially because Cetuximab binds to the EGFR receptor on the surface of 4T1 cells, allowing ^177^Lu to act on cells for a long time while free ^177^Lu is washed after 4 h.

Due to the presence of Cetuximab, ^177^Lu-3,4-HOPO-Cetuximab can specifically bind to EGFR-positive receptors in 4T1 cells, and the corresponding cell-binding rate increases as the duration of action increases. Since ^177^Lu cannot penetrate the cell membrane, the β-particles emitted during cell culture damage the cells, resulting in a decrease in cell proliferation ability. However, after washing with the medium, there is almost no ^177^Lu in the 4T1 cell culture system, and this is illustrated by the cell-binding assay. Since the cells only interacted with 500 kBq ^177^LuCl_3_ for 4 h, the damaged cells were gradually repaired, and the cell activity gradually increased with time, reaching 90.40% at 96 h, which was significantly increased compared with 82.10% at 8 h. The cells were treated with 500 kBq ^177^Lu-3,4-HOPO-Cetuximab for 8 h, ^177^Lu-3,4-HOPO-Cetuximab was retained in the cells by binding of the antibody Cetuximab to the EGFR receptor positively expressed on the surface of 4T1 cells, and sustained beta-ray damage Cells, causing cell activity to decrease over time.

Intravenous injection of ^177^Lu-3,4-HOPO-Cetuximab in the treatment of 4T1 nude mice xenografts showed that the weight of nude mice in the treatment group will decrease in a short period of time because of the strong growth of tumour cells at this stage and rapid consumption of nutrition in nude mice. Substance, after 7 days, showed the weight of nude mice in the treatment group began to rise, indicating that the tumour cell activity in nude mice decreased, and the metabolic capacity decreased. From the growth of transplanted tumour volume, the growth rate of xenografts in the treated group compared with the control group. As it slows down significantly, the growth curve slope prediction is expected to reach a negative value after a certain time, and it reaches a significant anti-tumour effect after 20 days. This series of data confirms the second part of ^177^Lu-3,4-HOPO-Cetuximab for 4T1 The killing effect of ^177^Lu *in vitro* and the targeting ability of Cetuximab. After 20 days of administration, the data of the radioactive distribution of the nude mice in the treatment group showed that the liver, tumour, and bone were the three organs with the highest radioactivity accumulation, and the ^177^Lu accumulation of ^177^Lu-3,4-HOPO-Cetuximab in the bone site was relatively low. The clinical case shows that ^177^Lu-labelled DOTA-coupled targeting material can cause severe bone metastasis after treatment for a period of time, *in vivo* radioactivity distribution data show that the dose deposition of HOPO in the liver and bone were lower than that of DOTA, and this indicates that HOPO has a better ability to promote the excretion of metal ions than DOTA. 3,4-HOPO is expected to become a new targeted therapy drug for clinical nuclear medicine to solve the problem of bone metastasis.

3,4-HOPO is a bidentate chelating agent synthesised by malt phenol as a template through a series of chemical synthesis steps. At present, the medical community has little research and application on HOPO chelate. Hagemann et al. (2017) showed that 3,2-HOPO coupled with the FGFR-2 receptor by the ^227^Th marker was visible in the *in situ* 4T1 model of invasive nude mice. The anti-tumour activity (Tinianow et al., 2016) and another independent synthesis of octadentate 3,2-HOPO-conjugated monoclonal antibody for ^89^Zr labelling both result in the *in vivo* stability being stronger than DTPA; in addition, there exists a report (Deri et al., 2015) on nuclear label HOPO research into the use of chelators since nuclear medicine developers have shown great potential. ^177^Lu-3,4-HOPO-Cetuximab has obvious killing and lethal effect on 4T1 tumours.

## Conclusions

We have confirmed that bidentate 3,4-HOPO has shown strong chelation application value, and it is necessary to synthesise four or more teeth 3,4-HOPO must have a more powerful clinical medicine role.

## Data Availability Statement

The raw data supporting the conclusions of this article will be made available by the authors, without undue reservation.

## Ethics Statement

The animal study was reviewed and approved by Soochow University.

## Author Contributions

KL was responsible for collecting data for cell and animal experiments and writing article. YZ and XW were responsible for the production and purification of molecular materials. RZ was responsible for data collation. CM and RH were responsible for proposing the experimental ideas and revising the article. All authors contributed to the article and approved the submitted version.

## Conflict of Interest

The authors declare that the research was conducted in the absence of any commercial or financial relationships that could be construed as a potential conflict of interest.

## Publisher's Note

All claims expressed in this article are solely those of the authors and do not necessarily represent those of their affiliated organizations, or those of the publisher, the editors and the reviewers. Any product that may be evaluated in this article, or claim that may be made by its manufacturer, is not guaranteed or endorsed by the publisher.
